# Association Between Apolipoprotein B and Coronary Artery Disease Among Hypertensive Patients: A Systematic Review of the Prospective and Retrospective Studies

**DOI:** 10.7759/cureus.49854

**Published:** 2023-12-03

**Authors:** Israa Nathir, Fatimatuzzahra Abd Aziz, Raid Hashim

**Affiliations:** 1 Discipline of Clinical Pharmacy, School of Pharmaceutical Sciences, Universiti Sains Malaysia, Penang, MYS; 2 Department of Pharmacy, Al-Rasheed University College, Baghdad, IRQ; 3 College of Pharmacy, Al-Farahidi University, Baghdad, IRQ

**Keywords:** coronary artery diseases, systematic review, normotensive, hypertensive, apolipoprotein b

## Abstract

The predictive value of apolipoprotein B (apo B) has been proven in the development of coronary artery disease (CAD) among normotensives only, but it has not been directly studied in hypertensive patients. The objective of this study is to explore the association between apo B and CAD among patients with hypertension. Search strategies were conducted on September 24, 2022, and involved the databases PubMed, Web of Science, and Scopus. The current systematic review included observational case-control and cohort study design involving adult humans, both hypertensives and normotensives. The selected studies were restricted to those written in the English language and published after 2000. Reviews, interventional, animal, and overlapping studies, grey literature, and articles without full text were excluded from the current study. The modified Newcastle-Ottawa Scale was used to assess the risk of bias for the screened studies after data extraction. Out of 3644 publications, only five studies were included in the review, including 5222 participants. Of those, 2335 were hypertensive, 733 of them developed CAD, and 296 normotensive subjects developed CAD. The average apo B was 1.09 g/l and 1.07 g/l for hypertensives and normotensives, respectively. The risk of developing CAD is higher in patients with hypertension, or those with higher apo B. Moreover, the risk of CAD was exacerbated in hypertensive participants with elevated apo B. This systematic review highlights the independent power of apo B on the development of CAD among both hypertensive and normotensive subjects.

## Introduction and background

Hypertension is the main risk factor for cardiovascular disease (CVD) and all-cause mortality over the world [1]. Hypertension is the major cause of atherosclerosis and it has damaging effects on endothelial cells [2]. A systematic review has shown that the effect of antihypertensive drugs on reducing coronary artery disease (CAD) was about 8-14%, which is still below 20-25% for the risk of prediction of CAD attributed to blood pressure. Thus, the lowering of elevated blood pressure alone is inadequate to eliminate the wholly CAD risk in hypertensive patients [3]. These findings advocate that the link between hypertension and CAD is complex and may include other factors together with the rise in blood pressure, such as abnormal body mass index, lipid disturbance, glucose intolerance, and hyperinsulinemia, which are usually interrelated, and explore independent predictors of both CAD and hypertension. Dyslipidaemia can be assessed by traditional lipid markers; however, they have insufficient preciseness [4]. On the other hand, protein-transporting molecules like apolipoprotein B (apo B) are present in all types of atherogenic lipoproteins including low-density lipoprotein cholesterol (LDL-C), very low-density lipoprotein cholesterol (VLDL-C), and intermediate-density lipoproteins (IDL), and their determination enables a more accurate estimation of atherogenicity than the traditional lipid biomarkers [5]. The risk of CAD has been proven to be predicted by apo B among normotensives, but it has not been studied widely in hypertensive patients. Previous studies indicate that ap B may be linked, via a variety of pathways, to the development of CAD in hypertensive patients. These pathways include the infiltration and retention of lipoproteins containing apo B in the arterial wall, the stimulation of endothelial dysfunction, and an increase in the severity of coronary artery stenosis [6]. The aim of this study is to explore the association between apo B and CAD among patients with hypertension. We present this article in accordance with the Preferred Reporting Items for Systematic Reviews and Meta-Analyses (PRISMA) reporting checklist.

## Review

Methods

A survey was done on PROSPERO to ensure the originality of the study.

Search of Literature

**Figure 1:**
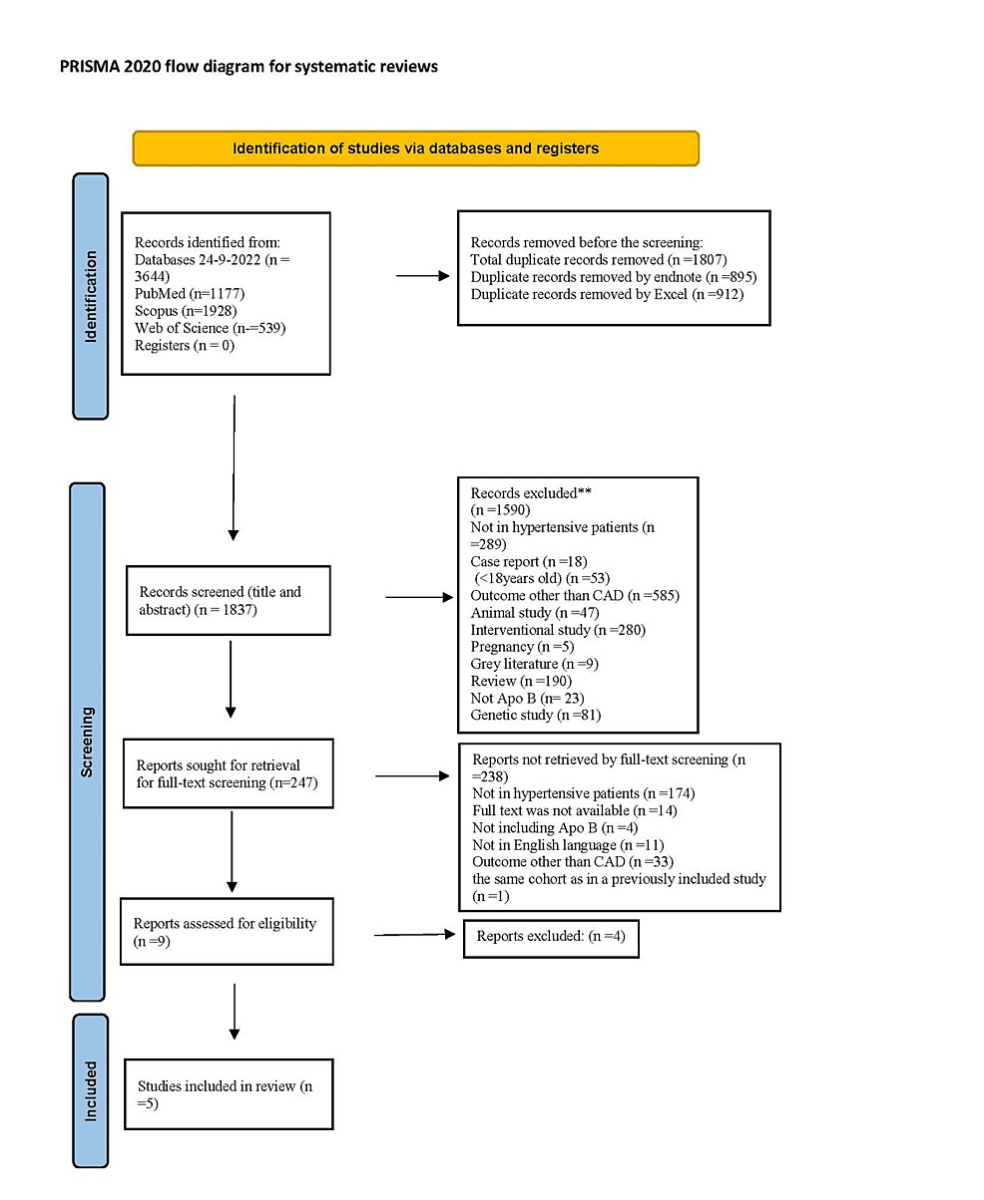
PRISMA flow diagram of the included studies in the systematic review PRISMA: Preferred Reporting Items for Systematic Reviews and Meta-Analyses

Search strategies on the databases PubMed, Web of Science, and Scopus were performed on September 24, 2022. The study protocol was prospectively registered at the International Prospective Register of Systematic Reviews (PROSPERO) at https://www.crd.york.ac.uk/prospero/display_record.php?ID=CRD42022369662. The inclusion criteria of the current study were observational case-control and cohort studies in which the primary outcome of interest has been reported, studies involving adult humans, and hypertensive and normotensive participants. The screening was restricted to publications in the English language and those published after the year 2000. Exclusion criteria were reviews, interventional, overlapping, and animal studies; grey literature; studies involving participants < 18 years old; and articles without full text. The literature search doesn’t include restrictions for gender, ethnicity, or socioeconomic status. The study was conducted in accordance with the PRISMA Statements 2020 for reporting the systematic review [7], and the PRISMA checklist is attached in the supplementary material 1. Key terms used were ((Apolipoproteins B) OR (Apolipoprotein-b)) AND (Hypertension) for PubMed search, TITLE-ABS-KEY (apolipoprotein AND b) AND (hyperten*) AND (LIMIT-TO (PUBSTAGE, "final")) AND (LIMIT-TO (DOCTYPE, "ar")) AND (LIMIT-TO (LANGUAGE, "English")) AND (LIMIT-TO (SRCTYPE, "j")) for Scopus search, and (Apolipoprotein b) and hyperten* refined by article documents and English language only for Web of Science search, search protocol explained in details in search strategy supplementary material 2. To remove duplicate papers, the resulting articles from each database were exported to the EndNote program in a separate group. After that, the duplicated articles were removed from all references section in the EndNote. Later, the articles were exported to an Excel sheet, which contained a number for each manuscript, name of the authors, year of publication, journal name, DOI, link, abstract, researcher 1 (Israa Nathir), inclusion/exclusion (which was filled as 0 or 1), reason (only for the excluded papers), then the researcher should mention the reason, researcher 2 (Fatimatuzzahra Abd Aziz), inclusion/exclusion, reason, researcher 3 (Raid D. Hashim), inclusion/exclusion, reason. Afterward, the articles were sorted alphabetically by title, and the duplicated papers were removed. The reasons for excluding the papers are mentioned in Figure 1. In the selected studies, hypertensive patients were identified by having an average of three or more readings of blood pressure, where systolic blood pressure is ≥140 mm Hg and diastolic blood pressure is ≥ 90 mm Hg for newly diagnosed patients or patients on antihypertensive drugs. Normotensive participants were identified as having an average systolic blood pressure of <140 mm Hg and a diastolic blood pressure of <90 mm Hg by three or more readings. The outcomes of CAD were identified as obstructive, non-obstructive coronary artery diseases, and spontaneous coronary artery dissection.

Selection Process

Title and abstract screening were performed by three independent researchers based on predefined criteria; discrepancies were solved by discussion between the researchers, and if the discussion didn’t solve the discrepancy, then a consult from an expert reviewer was considered (Abbas Abdulmueed Mustafa). Subsequently, full-text screening for eligibility was conducted, as all previously included papers were downloaded and those that had no available full text on the search database were excluded at once. The three independent researchers extracted data from eligible text using a standardized pilot form. Discrepancies were removed by mutual agreement or by the fourth reviewer. One study [8] was excluded during the data extraction process due to including the same baseline data of participants by the same author which was used previously in a different study included in the current systematic review [9].

Data Extraction

After full-text screening, data extraction was started for the selected studies which included the author name, year of publication, total sample size, number of participants with and without hypertension, country, study design, fasting state, period of follow-up, (age, number of males, females, smoking status, BMI, apo B, apo A, TC, TG, LDL-C, HDL-C, VLDL-C, and non HDL-C, number of participants developing diabetes mellitus, and CAD were reported for hypertensives and normotensives). The standard mean difference was executed for apo B, apo A, LDL-C, TC, TG, and HDL-C to unify the units of these variables as explained in supplementary materials 3 and 4. Data for all variables were reported from the included studies in terms of mean and standard deviation as there was no missing non-reported data.

Quality Assessment

The finally included papers were assessed for quality. The modified Newcastle-Ottawa Scale (NOS) was used for the quality assessment of observational (prospective and retrospective) studies included in the current systematic review. This quality assessment scale had three categories (parts); the selection part with 4 as a maximum score, the comparability part with 2 as a maximum score, and the exposure part with 3 as a maximum score [10]. Supplementary material 5 explains the criteria and the score of the modified NOS. The total score was calculated to consider those studies with scores of 7-9 as high quality and included in the current systematic review as explained in supplementary material 5. Studies scoring ≤ 6 were considered low-quality studies and were excluded. The robustness of the synthesized results is explained in supplementary material 6.

Results

Study Characteristics

Literature search and screening were performed according to PRISMA guidelines Figure 1. A total of 3644 publications were identified from the search database (PubMed, Scopus, and Web of Science) as explained in Figure 1. Eight hundred ninety-five duplicated papers were removed by EndNote, and then 912 duplicated papers were uninvolved by Excel. Subsequently, 1837 studies were further screened according to the title and abstract. From those, only 247 studies remained for the full-text screening, the reasons for excluding those papers were mentioned in Figure 1. The full-text screening was operated to give only nine eligible studies. After applying the modified NOS for quality assessment for the nine observational studies, three studies were excluded due to having a score of ≤ 6 as reported in supplementary material 5.

Patient Characteristics

 Five studies were included in the review which contained a total of 5222 participants, 2335 were hypertensive, and 733 of them developed CAD during the study period [9,11-13]. Two studies were conducted in China, one study was accomplished in Italy, and Turkey, and multicenter in the USA. Age and gender were reported in all of the included studies, smoking status was mentioned in two studies, BMI was reported in four manuscripts, and apo B, apo A, LDL-C, TC, TG, and HDL-C were measured in the five included studies as shown in supplementary material 7.

Socio-Demographic Data

Females were the most predominant participants in one study [14]. There was no significant difference in the number of male and female participants in the remaining studies. There was a significant difference in the mean age among hypertensives compared to normotensives in four of the five selected studies where hypertensive patients have shown higher mean age except in [9]. With respect to smoking status, normotensives have shown a significantly higher prevalence compared to hypertensives in only two of the selected studies. Body mass index was significantly lower in normotensive subjects in the five selected studies. Surprisingly only in one of the selected studies, there was a significant difference in mean serum apo B [10] where it was significantly higher in hypertensive patients. Apo A was significantly higher among normotensive subjects only in one study [9]. Total cholesterol was significantly higher in hypertensive subjects [10]. In addition, hypertensive females had higher TC in comparison to normotensive females [12,13]. On the other hand, the level of TC was comparable between the groups in the remaining studies. In 4 of the 5 studies, TG was significantly higher among hypertensive participants except in [13] as well and TG was significantly higher among hypertensive females compared to normotensive females [13]. Among hypertensive females, LDL-C was significantly higher compared to normotensive females [12,13] as the results of these two studies were stratified according to sex, and it was significantly higher in hypertensive patients compared to normotensive participants [11]. There was no significant difference among groups in terms of HDL-C, only [9] showed higher HDL-C among normotensive subjects when compared to those with hypertension. In addition, one study has shown a significantly higher HDL-C in normotensive compared to hypertensive females [12]. Only one of the selected studies mentioned that the prevalence of diabetes was significantly higher among hypertensive patients [13]. Development of CAD among participants was significantly higher among the hypertensive group [12,13]. Fadl revealed no significant difference between the two groups [14]. Another study [11] did not record any CAD during the study among the normotensive group. Sechi 2001 concluded that only 60 (15.4%) of 389 hypertensive subjects had developed CAD in the study period [9]. The characteristics of each study are explained in Table 1.

**Table 1:** Characteristics of each study Variables measured as mean ± standard deviation or as percentages, H: hypertensives, N: normotensives, apo B: apolipoprotein, apo A: apolipoprotein A, BMI: body mass index, LDL-C: low-density cholesterol, TC: total cholesterol, TG: triglyceride, HDL-C: high-density cholesterol, DM: diabetes mellitus, CAD: coronary artery disease.

Name of the first author	Wang H [11]	Sechi LA [9]	Onat A [12]	Yang SH [13]	Fadl YY [14]		
Year of publication	2017	2001	2008	2016	2015		
Fasting status	Yes	yes	yes	yes	yes		
Sample size	349	712	3034	805	1045		
Patients with (H)	SDH 92	IDH 45	ISH 80	389	787	481	461
normotensives	132	323	1526	324	582		
Country	China	Italy	Turkey	China	Multicenter USA		
Study design	Cross-sectional	Cross-sectional	prospective survey	Cross-sectional	prospective		
Period of follow up	No	No	6.6 year	No	2 years		
Age (year) of (H)	47.54±12.29	43.51±10.44	56±(36.5-72)	54±12	53.5±12.1	56.02±9.58 m 61.3±9.89 f	61.7±2
Age (year) of (N)	37.5(28-46)	52±14	42.6±10.4	53.44±11.08 m 55.08±9.99 f	56.8±2		
Number of males (H)	56(60.86%)	20(44.4%)	48(60%)	206	683	314	69
Number of males (N)	73(55.3) %	165	425	206	81		
Smoking (H)				202(65.6%)	21		
Smoking (N)				127(67.7%)	26		
BMI (H)	27.74± 3.74	27.06±3.59	25.6(23.87-28.75)		25.6(23.87-28.75)	26.65±3.33m 25.59±3.56f	28.6±0.9
BMI (N)	25.6(23.87-28.75)		28.9	24.97±3.44m 24.21±3.08f	27.6±0.6		
Apo B (H)	1.03(0.87-1.21)	0.99(0.77-1.09)	0.98(0.8-1.14)	1.23±0.32	110.9m 111f	1±0.27m 1.07±0.26f	1.22±0.29
Apo B (N)	0.87(0.72-1)	1.22±0.32	105.5m 101f	1.03±0.31m 1.07±0.33f	1.23±0.28		
Apo A (H)	1.37(1.27-1.51)	1.34(1.2-1.45)	1.31(1.2-1.45)	1.49±0.29	134.4m 147.3f	1.31±0.26m 144±0.31f	1.18±0.25
Apo A (N)	1.13(1.17-1.48)	1.55±0.38	130.4m143.5 143.5f	1.3±0.26m 1.46±0.28	1.18±0.25		
LDL-C (H)	3.11(2.75-3.54)	2.74(2.35-3.34)	3(2.62-3.58)	3.52±0.96	115.5M 122.3 F	3.18±0.93M 3.4±0.91F	3.13±0.98
LDL-C (N)	2.53(2.05-3.19)	3.38±0.91	115.3M 115.6 F	3.28±1.04M 3.66±1.26F	3.08±0.98		
TC (H)	5(4.52-5.75)	4.8(4.25-5.3)	5(4.4-5.48)	5.38±1.11	191.8 M 202.5 F	4.79±1.03M 5.04±0.94 F	5.1±1.17
TC (N)	4.6(4-5.57)	5.33±0.98	187.5 M 188.9 F	4.83±1.11M 5.3±1.28 F	5.13±1.11		
TG (H)	1.89(1.37-2.89)	1.8(1.15-3.39)	1.7(1.03-2.3)	1.46±0.98	177 M 157.5 F	1.67(1.23,2.38) M 1.69(1.26,2.44)F	2.16±1.27
TG (N)	1.2(0.96-1.86)	1.29±0.58	147.3 M 128.7 F	1.63(1.12,2.31) M 1.59(1.07,2.2)F	2.36±1.37		
HDL-C (H)	1.05(0.97-1.24)	1(0.92-1.2)	1.02(0.96-1.22)	1.32±0.39	39.5M 46.2 F	1.03±0.29M 1.16±0.34F	1.04±0.31
HDL-C (N)	1.06(1-1.27)	1.4±0.4	39.4 M 47.1F	1.03±0.29M 1.23±0.41F	1.01±0.28		
DM (H)	13(14.13%)	6.(13.33%)	10(12.5%)				27
DM (N)					13		
H with CAD	22(23.9%1)	8(17.78%)	16(20%)	60	192(18.1%)	244(78%)M 102(63%)F	89(19.3%)
N with (N)o CAD			46(4.7%)	137(66.8%)M 43(36.4%)F	113(19.4%)		

Discussion

Over the decades, factors that contributed to the development of CAD ‎have been thoroughly studied. Hypertension and dyslipidemia were among the factors ‎that have the highest predictive role in the development of CAD. The pathophysiological ‎role of apo B in the development of CAD has received much more attention during the ‎last years in an attempt to explain the higher incidence of CAD in certain populations ‎with normal lipid profiles. Most studies have investigated this role distinctly ‎from concurrent hypertension. Only a minority of studies have taken into consideration ‎the plasma concentration of apo B when studying the correlation between hypertension ‎and the development of CAD. ‎One [12] of the 5 studies has shown significantly higher apo B concentration in hypertensive ‎patients compared to the normotensive. In addition, it might be surprising that only 2 of the reviewed studies [12,13] have shown a significant increase in the development of CAD in ‎hypertensive patients compared to normotensive. Of these two studies, apo B was ‎significantly higher in hypertensive patients compared to the normotensive in only 1 ‎study [12]. Interestingly, 2 of the reviewed studies [11] have shown a comparable percentage of ‎occurrence of CAD in both hypertensive and normotensives concurrently with ‎statistically insignificant differences in mean serum apo B between the two groups. These ‎results highlight the independent impact of apo B on the development of CAD. ‎Furthermore, even when comparing within the same hypertensive group, one of the five ‎studies has revealed a significantly higher rate of occurrence of CVD in the subgroup with ‎higher mean apo B concentration [9].‎

‎Three of the included studies were cross-sectional [9,11,13], and only 2 were prospective studies [12]. Three of the screened studies [15-17] were excluded due to lacking a control group of participants without hypertension although they were compatible with the required criteria. One study [8] did not account for the number of CAD in the control group and this might make the comparison difficult and might be considered a source of heterogeneity, this might be a limitation to the evidence included in the review. A further limitation that we have observed in certain studies was in the diagnosis of hypertensive subjects, which depends on three readings within 1 day and this might lead to an overestimation in the prevalence of hypertension among the selected participants. Another limitation is the use of antihypertensive therapy among certain patients included in the selected studies. The use of antihypertensive therapy had a greater impact on the outcome of CAD, consequently, this might affect our conclusion.

Although the current literature-based systematic review has provided the most comprehensive assessment of apo B and the risk of CAD among hypertensive patients, however, there was a limited number of published research that are directly studying the correlation between apo B and CAD among hypertensive patients. The exclusion of grey literature such as thesis, government publications, unpublished research, seminar journals, and conference papers which might have studies with null or negative results compared to the hypothesis of the current study might be one of the limitations for the evidence supported by this systematic review and a source of publication bias. The predictive value of apo B for CAD has already been proved [18-21] and the use of apo B as a predictor for the development of CAD should be encouraged especially for hypertensive patients due to their high risk of developing CAD. Furthermore, laboratory test for apo B is broadly available, automated, and standardized, and they can be conducted on both fresh and frozen blood specimens as well as it does not require fasting status like in other lipid profiles [22]. Two studies [10,13] revealed associations between apo B and the development of CVD in the normotensive group only and this comes in line with previous studies confirming apo B as a predictor of CVD. More research is required to confirm this relationship specifically among hypertensive populations taking into consideration the limitations mentioned earlier. 

## Conclusions

The risk of developing CAD is significantly increased in patients with hypertension compared to normotensive patients. The risk of developing CAD is significantly increased in patients with a higher plasma apo B concentration. The risk of developing CAD is minimized in patients with hypertension compared to normotensives when plasma apo B concentration is comparable between the two groups. The risk of the development of CAD is aggravated in hypertensive patients with higher plasma concentrations of apoB.

This systematic review highlights the independent power of apo B on the development of CVD among both hypertensive and normotensive subjects.

## Appendices

**Table 2:** PRISMA checklist (Supplementary material 1) PRISMA: Preferred Reporting Items for Systematic Reviews and Meta-Analyses

Section/topic	Item No	Checklist item	Reported on Page Number/Line Number	Reported on Section/Paragraph
TITLE				
Title	1	Identify the report as a systematic review.	P1, L1	Title
ABSTRACT				
Abstract	2	See the PRISMA 2020 for Abstracts checklist (Table 2).	P1, (L1-L22)	Abstract page
INTRODUCTION				
Rationale	3	Describe the rationale for the review in the context of existing knowledge.	P2, (L1-L17)	S1
Objectives	4	Provide an explicit statement of the objective(s) or question(s) the review addresses.	P2, (L18-L19)	S1
METHODS				
Eligibility criteria	5	Specify the inclusion and exclusion criteria for the review and how studies were grouped for the syntheses.	P2, (L26-L30)	S3
Information sources	6	Specify all databases, registers, websites, organisations, reference lists and other sources searched or consulted to identify studies. Specify the date when each source was last searched or consulted.	P2, (L21-L22), Figure 1	S2
Search strategy	7	Present the full search strategies for all databases, registers and websites, including any filters and limits used.	P3, (L1-L7), Table 2 supp material	S1
Selection process	8	Specify the methods used to decide whether a study met the inclusion criteria of the review, including how many reviewers screened each record and each report retrieved, whether they worked independently, and if applicable, details of automation tools used in the process.	P3, (L24-L27)	S4
Data collection process	9	Specify the methods used to collect data from reports, including how many reviewers collected data from each report, whether they worked independently, any processes for obtaining or confirming data from study investigators, and if applicable, details of automation tools used in the process.	P3, (L27-L30)	S4
Data items	10a	List and define all outcomes for which data were sought. Specify whether all results that were compatible with each outcome domain in each study were sought (e.g. for all measures, time points, analyses), and if not, the methods used to decide which results to collect.	P3, (L19-L24)	S3
	10b	List and define all other variables for which data were sought (e.g. participant and intervention characteristics, funding sources). Describe any assumptions made about any missing or unclear information.	P3, (L19-L24)	S3

Study risk of bias assessment	11	Specify the methods used to assess risk of bias in the included studies, including details of the tool(s) used, how many reviewers assessed each study and whether they worked independently, and if applicable, details of automation tools used in the process.	P4, (L6-L12), Table 5 supp material	S 2. Supp material
Effect measures	12	Specify for each outcome the effect measure(s) (e.g. risk ratio, mean difference) used in the synthesis or presentation of results.	(P3,L34 - P4,L3)Table 3,4 supp material	S 4, Page3 – S1, Page4
Synthesis methods	13a	Describe the processes used to decide which studies were eligible for each synthesis.	Table 1, Table 7 supp material	Page 9, 10. Supp material
	13b	Describe any methods required to prepare the data for presentation or synthesis, such as handling of missing summary statistics, or data conversions.	Table 3, 4 supp material	Supp material
	13c	Describe any methods used to tabulate or visually display results of individual studies and syntheses.	Table 1, Table 7 supp material	Page 9, 10. Supp material
	13d	Describe any methods used to synthesize results and provide a rationale for the choice(s). If meta-analysis was performed, describe the model(s), method(s) to identify the presence and extent of statistical heterogeneity, and software package(s) used.	N/A	
	13e	Describe any methods used to explore possible causes of heterogeneity among study results.	N/A	
	13f	Describe any sensitivity analyses conducted to assess robustness of the synthesized results.	P4,( L15-L16), Table 6 supp material	Last section . Supp material
Reporting bias assessment	14	Describe any methods used to assess risk of bias due to missing results in a synthesis (arising from reporting biases).	N/A	
Certainty assessment	15	Describe any methods used to assess certainty (or confidence) in the body of evidence for an outcome.	N/A	
RESULTS				
Study selection	16a	Describe the results of the search and selection process, from the number of records identified in the search to the number of studies included in the review, ideally using a flow diagram.	P4, (L17-L19), Figure 1	S 2
	16b	Cite studies that met many but not all inclusion criteria (‘near-misses’) and explain why they were excluded.	Table 3,4,6 supp material, Figure1	
Study characteristics	17	Cite each included study and present its characteristics.	Table 1,7 supp material	
Risk of bias in studies	18	Present assessments of risk of bias for each included study.	Table 6 supp material	
Results of individual studies	19	For all outcomes, present, for each study: (a) summary statistics for each group (where appropriate) and (b) an effect estimate and its precision (e.g. confidence/credible interval), ideally using structured tables or plots.	Table 7 supp material	

Results of syntheses	20a	For each synthesis, briefly summarise the characteristics and risk of bias among contributing studies.	Table 1 (P10,11,12)	
	20b	Present results of all statistical syntheses conducted. If meta-analysis was done, present for each the summary estimate and its precision (e.g. confidence/credible interval) and measures of statistical heterogeneity. If comparing groups, describe the direction of the effect.	(P6, L10 - P7, L4)	S 2, section 1
	20c	Present results of all investigations of possible causes of heterogeneity among study results.	N/A	
	20d	Present results of all sensitivity analyses conducted to assess the robustness of the synthesized results.	N/A	
Reporting biases	21	Present assessments of risk of bias due to missing results (arising from reporting biases) for each synthesis assessed.	N/A	
Certainty of evidence	22	Present assessments of certainty (or confidence) in the body of evidence for each outcome assessed.	N/A	
DISCUSSION				
Discussion	23a	Provide a general interpretation of the results in the context of other evidence.	P7, (L6 - L13)	S 2
	23b	Discuss any limitations of the evidence included in the review.	P7, (L28 - L29)	S 3
	23c	Discuss any limitations of the review processes used.	(P7, L30 - P8, L6)	P7, S 3. P8,S 1
	23d	Discuss implications of the results for practice, policy, and future research.	P8(L6 - L10)	S 1
OTHER INFORMATION				
Registration and protocol	24a	Provide registration information for the review, including register name and registration number, or state that the review was not registered.	The study protocol was prospectively recorded in the international prospective register of systematic reviews (PROSPERO) by https://www.crd.york.ac.uk/prospero/display_record.php?ID=CRD42022369662	
	24b	Indicate where the review protocol can be accessed, or state that a protocol was not prepared.	The review can be accessed	
	24c	Describe and explain any amendments to information provided at registration or in the protocol.	Odds ratio and risk ratio were not considered in the current review	
Support	25	Describe sources of financial or non-financial support for the review, and the role of the funders or sponsors in the review.	N/A	
Competing interests	26	Declare any competing interests of review authors.	The author declares any conflict of interest	
Availability of data, code and other materials	27	Report which of the following are publicly available and where they can be found: template data collection forms; data extracted from included studies; data used for all analyses; analytic code; any other materials used in the review.	Table 2,3,4,5,6,7 supp material and Table 1	

**Table 3:** Search strategy (Suplementaty material 2)

	N	Search date	Database	Search Term
1	24-9-2022		PubMed	((Apolipoproteins B) OR (Apolipoprotein-b)) AND (Hypertension)
2	24-9-2022		Scopus	TITLE-ABS-KEY (apolipoprotein AND b ) AND ( hyperten*) AND ( LIMIT-TO ( PUBSTAGE , "final" ) ) AND ( LIMIT-TO ( DOCTYPE , "ar" ) ) AND ( LIMIT-TO ( LANGUAGE , "English" ) ) AND ( LIMIT-TO ( SRCTYPE , "j" ) )
3	24-9-2022		WOS	(Apolipoprotein b) and hyperten* refined by article documents and English language only

**Table 4:** Mean difference calculation for Apo B and Apo A (g/l) (Supplementary material 3) H: Hypertensive, N: Normotensive, Apo B: apolipoprotein B, Apo A: apolipoprotein A

Name of the first author	Apo B H	Apo B N	Apo A H	Apo A N
Wang HH, Y [11]	1.03	0.87	1.37	1.31
	0.99		1.34	
	0.98		1.31	
Sechi LA [9]	1.23	1.22	1.49	1.55
Onat A [12]	1.109 M	1.05 M	1.344 M	1.304 M
	1.11 F	1.01 F	1.473 F	1.435 F
Yang SH [13]	1 M	1.03 M	1.31 M	1.3 M
	1.07 F	1.07 F	1.44 F	1.46 F
Fadl YY [14]	1.22	1.23	1.18	1.18

**Table 5:** Mean difference calculations for LDL-C, TC, TG, and HDL-C (mmol/l) (Supplementary material 4) N: Normotensive patients, H: hypertensive subjects, LDL-C: low-density lipoprotein cholesterol, TC: total cholesterol, TG: triglyceride, HDL-C: high-density lipoprotein cholesterol.

Name of the first author	LDL-C H	LDL-C N	TC H	TC N	TG H	TG N	HDL-C H	HDL-C N
Wang H [11]	3.11	2.53	5	4.6	1.89	1.2	1.05	1.06
	2.74		4.8		1.8		1	
	3		5		1.7		1.02	
Sechi LA [9]	3.52	3.38	5.38	5.33	1.46	1.29	1.32	1.4
Onat A [12]	2.98 M	2.97 M	4.95 M	4.84 M	1.99 M	1.66 M	1.02 M	1.02 M
	3.16 F	2.98 F	5.23 F	4.88 F	1.77 F	1.45 F	1.19 F	1.21 F
Yang SH [ 13]	3.18 M	3.28 M	4.79 M	4.83 M	1.67 M	1.63 M	1.03 M	1.03 M
	3.4 F	3.66 F	5.04 F	5.3 F	1.69 F	1.59 F	1.16 F	1.23 F
Fadl YY [14]	3.13	3.08	5.1	5.1	2.16	2.36	1.04	1.01

**Table 6:** Modified NOS criteria for the screened studies after data extraction (Supplementary material 5) NOS: Newcastle–Ottawa Scale

Case-control		
Selection, maximum score (4)		Score
1. Is the case definition adequate?	a) yes, with independent validation ¨e.g. ICD codes in the database or self-report with no reference to primary record or no description, >1 person/record/time/process to extract information or reference to primary record sources such as x-rays or medical/hospital records	1
	b) yes, eg record linkage or based on self-reports	
	c) no description	
2. Representativeness of the cases	a) consecutive or obviously representative series of cases ¨	1
	b) potential for selection biases or not stated	
3. Selection of Controls	a) community controls (outpatients normotensive) ¨	1
	b) hospital controls (inpatients normotensive)	
	c) no description	
4. Definition of controls	a) no history of the disease (no initial CAD) ¨	1
	b) no description of the source	
Comparability, maximum score (2)		Score
1. Comparability of cases and controls on the basis of the design or analysis	a) the study represented normotensive as a control separated group ¨	1
	b) the study control group reported the same demographic and clinical characteristics as the case group¨	1
Exposure, maximum (3)		Score
1. Ascertainment of exposure	a) secure records (eg medical records) ¨	1
	b) structured interview where blind to case/control status ¨	1
	c) interview not blinded to case/control status	
	d) written self-report or medical record only	
	e) no description	
2. Same method of ascertainment for cases and controls	a) yes ¨	1
	b) no	
3. Non-Response Rate	a) same rate for both groups ¨	1
	b) non respondents described	
	c) rate different and no designation or not stated (non-respondent with no reasons)	
Cohort study		
Selection, maximum score (4)		Score
1. Representativeness of the exposed cohort	a) all hypertensive patients attending the hospital ¨	1
	b) random selection of hypertensive patients from the same hospital ¨	1
	c) non-random selection of hypertensive patients attending the hospital	
	d) no description of how the hypertensive patients were selected	
2. Selection of the normotensive cohort	a) drawn from the same hospital as the hypertensive patients¨	1
	b) drawn from a different source	
	c) no description of how the normotensives were selected	
3. Ascertainment of exposure	a) secure records (obtained from the medical records) ¨	1
	b) obtained by the laboratory examination during the research time¨	
	c) written self-report	
	d) no description	
4. Demonstration that outcome of interest was not present at start of the study	a) yes¨	1
	b) no	
Comparability, maximum score (1)		Score
1. Comparability of cohorts on the basis of the design or analysis	a) study reported hypertensive and normotensive participants as a separate group¨	1
	b) reported additional clinical and demographic characteristics among normotensives	
Outcome, maximum score (3)		Score
1. Assessment of outcome	a) independent blind assessment or secured medical record mentioned in the paper¨	1
	b) record linkage ¨	1
	c) self-report	
	d) no description	
2. Was follow-up long enough for outcomes to occur	a) yes (follow-up period for the outcome of interest mentioned clearly) or no need for follow-up for cross-sectional studies¨	1
	b) no	
3. Adequacy of follow-up of cohorts	a) complete follow-up - all subjects accounted for CAD¨	1
	b) subjects lost to follow-up unlikely to introduce bias a small number lost = >95 % follow-up, or description of those lost) ¨	1
	c) follow-up rate < 95% (select an adequate %) and no description of those lost	
	d) no statement	

**Table 7:** Modified NOS scores for the eligible studies included (Supplementary material 6) NOS: Newcastle–Ottawa Scale

First author		Selection (max4)	Comparability (max2)	Outcome/ Exposure (max3)	Total score
1	Wang HH, Y [11]	4	2	2	8
2	Zhao XX [17]	3	0	3	6
3	Sechi LA [9]	4	2	3	9
4	Onat A [12]	4	2	3	9
5	Yang SH, Y [13]	4	2	3	9
6	Fadl YY [14]	3	2	3	8
7	Sung JH [15]	3	0	3	6
8	Kim CW [16]	4	0	2	6

**Table 8:** Baseline characteristics of participants (Supplementary material 7) N: number, BMI: body mass index, Apo B: apolipoprotein B, apo A: apolipoprotein A, LDL-C: low-density lipoprotein cholesterol, TC: total cholesterol, TG: triglyceride, HDL-C: high-density lipoprotein sterol, DM: diabetes mellitus, CAD: coronary artery disease.

Variables	Studies N	Participants N	Among hypertensive	Among normotensive
Age	5	5222	54.19 years/ 2335	48.46 years/ 2887
Male	5	5222	1396/ 2335	950/ 2887
Smokers	2	1848	223/ 942	252/ 906
BMI	4	4510	26.6 kg/m^2^ / 1946	26.25 kg/m^2 ^/ 2564
Apo B	5	5222	1.09 g/l / 2335	1.07 g/l / 2887
Apo A	5	5222	1.36 g/l / 2335	1.33 g/l / 2887
LDL-C	5	5222	3.13mmol/l/ 2335	3.04 mmol/l / 2887
TC	5	5222	5 mmol/l / 2335	4.9 mmol/l / 2887
TG	5	5222	1.79 mmol/l / 2335	1.58 mmol/l / 2887
HDL-C	5	5222	1.08 mmol/l / 2335	1.15 mmol/l / 2887
DM	1	1045	56/ 461	13/ 582
CAD	5	5222	733/ 2335	296/ 2887
